# The differential diagnosis of medical and psychogenic disease in primary care

**DOI:** 10.1002/jgf2.661

**Published:** 2023-11-29

**Authors:** Kosuke Ishizuka, Kiyoshi Shikino, Yu Li, Daiki Yokokawa, Tomoko Tsukamoto, Yasutaka Yanagita, Jumpei Kojima, Shiho Yamashita, Kazutaka Noda, Takanori Uehara, Masatomi Ikusaka

**Affiliations:** ^1^ Department of General Medicine Chiba University Hospital Chiba Japan; ^2^ Department of General Medicine Yokohama City University School of Medicine Yokohama Japan; ^3^ Department of Community‐oriented Medical Education, Graduate School of Medicine Chiba University Chiba Japan; ^4^ Department of Medical Education, Graduate School of Medicine Chiba University Chiba Japan

## Abstract

Diagnosis and management of psychogenic diseases such as conversion disorder, somatic symptom disorder (SSD), illness anxiety disorder, falsehood disorder, and psychotic disorder require an elaborate biopsychosocial approach and are often challenging. Herein, we propose the following points to differentiate medical diseases from these psychogenic diseases: correspondence between symptoms and objective findings or activities of daily living (ADL) impairment; placebo effect; clear provocative or palliative factors; progressive time course; paroxysmal or intermittent symptoms; unfamiliar but not strange expressions; symptoms worsen during sleep or rest.

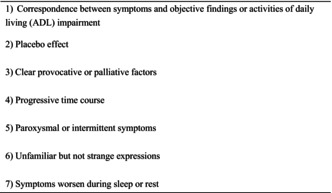

To the Editor,

Patients with psychogenic disease who present with physical symptoms are common, comprising approximately 30% of primary care outpatients.^1^ However, medically unexplained physical symptoms (MUPS) must be carefully distinguished from organic diseases, because making a diagnosis solely based on immediate recall may lead to missing a potential organic disease. Among psychogenic diseases, mood and anxiety disorders as well as schizophrenia may be considered pathological conditions similar to organic diseases (e.g., endocrine disorders) because of their response to medical treatment. However, diagnosis and management of psychogenic diseases such as conversion disorder, somatic symptom disorder (SSD), illness anxiety disorder, falsehood disorder, and psychotic disorder require an elaborate biopsychosocial approach and are often challenging.^2^ The five‐item A‐MUPS (Analgesics ineffective, Mental disorder history, Unclear provocative/palliative factors, Persistence without cessation, and Stress feelings/episodes) score can be used to differentiate between medical disease and SSD in patients with nonacute pain.^3^ The following points meticulously chosen based on the Diagnostic and Statistical Manual of Mental Disorders, Fifth Edition (DSM‐5) and A‐MUPS are presented to differentiate medical diseases from the latter psychogenic diseases (Table 1).^2^, ^3^

**TABLE 1:** The differential diagnosis of medical and psychogenic disease.

(1) Correspondence between symptoms and objective findings or activities of daily living (ADL) impairment
(2) Placebo effect
(3) Clear provocative or palliative factors
(4) Progressive time course
(5) Paroxysmal or intermittent symptoms
(6) Unfamiliar but not strange expressions
(7) Symptoms worsen during sleep or rest

## 1 CORRESPONDENCE BETWEEN SYMPTOMS AND OBJECTIVE FINDINGS OR ACTIVITIES OF DAILY LIVING (ADL) IMPAIRMENT

The possibility of psychogenic disease is higher when there is a discrepancy between the patient's medical history and physical examination, or between symptoms and ADL impairment.^2^

## 2 PLACEBO EFFECT

Analgesics produce pain relief not only through pharmacological effects but also through placebo effect.^3^ The placebo effect occurs when patients expect improvement of their symptoms. If the placebo effect is absent, a situation wherein somatic symptomatology is enhanced is more likely, and the symptoms may be subconscious expression that the patient does not want to be cured.^3^

## 3 CLEAR PROVOCATIVE OR PALLIATIVE FACTORS

The presence of clear provocative or palliative factors suggests medical disease.^3^ However, if these factors are unclear, psychogenic disease is more likely.^3^ In psychogenic diseases, functional and cortical issues evoke or strengthen the symptoms, and lack of pain signals from somatic organs make provocative or palliative factors unclear.^3^

## 4 PROGRESSIVE TIME COURSE

If symptoms progress, the possibility of medical disease should be considered. However, in psychogenic diseases, symptoms can be appealing to patients; hence, it is important to judge the course of the disease based on objective medical history such as interference with ADL, frequency of medication use, and patient behavior.^3^

## 5 PAROXYSMAL OR INTERMITTENT SYMPTOMS

While medical diseases have postures and times when they do not feel symptoms, patients with psychogenic diseases tend to complain of constant symptoms.^3^ As such patients tend to selectively focus on physical symptoms, unceasing consciousness of their pain would be causative.^3^

## 6 UNFAMILIAR BUT NOT STRANGE EXPRESSIONS

If the symptom is unfamiliar but not strange, the possibility that it is a highly specific symptom should be considered. For example, “air in the urine” (pneumaturia) may be caused by colovesical fistula. Whereas, symptoms such as “electromagnetic radiation” or “earthworms” raise the possibility of psychogenic disease or psychotic disorder.^4^

## 7 SYMPTOMS WORSEN DURING SLEEP OR REST

In psychogenic diseases, symptoms are relieved when stress load is reduced during sleep or rest.^5^ Conversely, when symptoms worsen under these conditions, the possibility of medical disease should be considered.^5^

By applying these pointers, we hope to help in differentiating between medical and psychogenic diseases in primary care. Further research is needed to verify external validations about these pointers.

## AUTHOR CONTRIBUTIONS

All authors had access to the data and a role in writing the manuscript.

## FUNDING INFORMATION

None.

## CONFLICT OF INTEREST STATEMENT

None.
